# Web-based interventions for prevention and treatment of perinatal mood disorders: a systematic review

**DOI:** 10.1186/s12884-016-0831-1

**Published:** 2016-02-29

**Authors:** Eleanor W. Lee, Fiona C. Denison, Kahyee Hor, Rebecca M. Reynolds

**Affiliations:** Tommy’s Centre for Maternal and Fetal Health, University of Edinburgh, Queen’s Medical Research Institute, 47 Little France Crescent, Edinburgh, EH16 4TJ Scotland; Obstetrics and Gynaecology, Queen Elizabeth University Hospital, 1345 Govan Road, Glasgow, G51 4TF Scotland; Endocrinology Unit, University/British Heart Foundation Centre for Cardiovascular Science, University of Edinburgh, Queen’s Medical Research Institute, 47 Little France Crescent, Edinburgh, EH16 4TJ Scotland

**Keywords:** CBT, Web-based, Perinatal, Depression, Pregnancy

## Abstract

**Background:**

Perinatal depression is strikingly common with a prevalence of 10–15 %. The adverse effects of perinatal depression on maternal and child health are profound with considerable costs. Despite this, few women seek medical attention. E-health, providing healthcare via the Internet is an accessible and effective solution for the treatment of depression in the general population. We aimed to conduct a systematic review of web-based interventions for the prevention and treatment of mood disorders in the perinatal period, defined as the start of pregnancy to 1 year post-partum.

**Methods:**

Six databases were searched until 26^th^ March 2015. Two researchers independently screened articles for eligibility. Of the 547 screened articles, four met the inclusion criteria. These included three randomised-controlled trials and one feasibility trial, with total data from 1274 participants. MOOSE and PRISMA guidelines were adhered to for the conduct and reporting of the systematic review.

**Results:**

All studies were conducted in the post-partum period. All reported an improvement in maternal mood following intervention. A significant improvement in depressive symptoms was measured using validated rating scales, such as the Edinburgh Postnatal Depression Scale (EPDS), either at post-treatment or follow-up which ranged from 3 to 12 months post study completion. For the two RCTs utilising the EPDS, the EPDS score reductions were (mean ± SEM) 8.52 ± 0.22 (Range 19.46 to10.94) and 9.19 ± 0.63 (Range, 20.24 to 11.05) for treatment groups and 5.16 ± 0.25 (Range 19.44 to 14.28) and 6.81 ± 0.71 (Range 21.07 to 14.26) for comparator groups. However attrition within studies ranged from 13 to 61 %. One study was rated as ‘good’ quality.

**Conclusions:**

Preliminary data suggests web-based therapies for perinatal depression delivered in the post-partum period may play a role in improving maternalmood but more studies are needed, particularly with interventions delivered antenatally. Further research is needed to address the limitations of the existing evidence base.

**Electronic supplementary material:**

The online version of this article (doi:10.1186/s12884-016-0831-1) contains supplementary material, which is available to authorized users.

## Background

Depression during the perinatal period is common, occurring in 10–15 % of pregnancies [1, 2], with the prevalence of depression peaking at 3 months postpartum. The adverse effects of perinatal depression on maternal and child health are profound [3–6] with estimated costs to the UK of £8.1 billion per year [7]. The *Saving Mothers' Lives Report* (2011) identified 29 women who died as a result of suicide between 2006 and 2008 [8]. Of these 29 women, 6 of them (21 %) had an underlying diagnosis of severe depressive illness. Despite this, few women seek professional advice due to physical and attitudinal barriers, long-waiting times for face-to-face psychotherapies, social stigma [9–13] and uncertainties about the potential risks of pharmacological therapies during pregnancy [14–16]. There is therefore an urgent need for a validated, effective, easily accessible, low-cost, non-pharmacological psychosocial evidence-based intervention to prevent or treat perinatal depression.

Computerised Cognitive Behavioural Therapy (CCBT) has proved an acceptable and effective treatment for depression in non-pregnant individuals [17–26] with a comprehensive meta-analysis demonstrating significant superiority compared to comparator therapies in the general population [18]. Based on this evidence, CCBT is one of the low-intensity psychosocial interventions that the National Institute for Health and Care Excellence (NICE) (which provides national guidelines for delivery of healthcare in England and Wales in the UK) recommends for treating persistent subthreshold depressive symptoms or mild to moderate depression in non-pregnant individuals [27].

NICE also suggests that CCBT should be offered to treat perinatal depression [28]. This is despite the scarcity of psychosocial interventions specifically designed to treat perinatal depression (particularly that originating antenatally) and the changing and specific psychosocial needs of women during pregnancy and post-partum [29, 30]. Further, women are increasingly using the Internet as a source of health information during pregnancy. For example, in a study of 613 women, almost 50 % used the Internet to enhance their involvement in pregnancy-related decision-making [31] and 90 % felt that health professionals should actively suggest suitable Internet sites to guide their further reading during pregnancy [31]. A CCBT resource for the self-management of perinatal depression could therefore represent an efficacious, accessible and economically sound resource [32]. CCBT also provides a level of anonymity not possible in clinical care, can be accessed at a time and place suitable for the participant and can be adapted to different cultures, languages and literacy levels to ensure the inclusion of vulnerable groups [33]. However, before CCBT is embedded in clinical practice for treatment of perinatal depression, there is a need for a systematic review of the existing evidence to determine whether web-interventions for pregnant and post-partum women are efficacious for prevention and treatment of perinatal mood disorders and potentially to inform the design of future studies if gaps in the current evidence base are identified.

## Methods

### Data sources

The Meta-analysis of Observational Studies in Epidemiology (MOOSE) guidelines were followed for the conduct [34], and the Preferred Reporting Items for Systematic Reviews and Meta-analyses (PRISMA) guidelines for the reporting [35] of this systematic review. The literature search was conducted using MEDLINE, PsycINFO, Embase and Cumulative Index to Nursing and Allied Health Literature (CINAHL) from inception until March 26^th^ 2015. The searches were limited to human studies and used terms as both keywords and indexing terms (Medical Subject Headings, MeSH) to identify studies related to depression AND pregnancy AND therapy AND online: ‘depression’, ‘depressive’, ‘depressed’, ‘mental-health’, ‘well-being’, ‘post-natal depression’, ‘pregnancy’, ‘perinatal care’, ‘pre-natal/ante-natal care’, ‘post-partum/post-natal care’, ‘pregnancy complication’, ‘self-help’, ‘cognitive behavioural therapy’, ‘behavioural therapy’, ‘psychotherapy’, ‘internet’, ‘online’, ‘online therapy’, ‘computer program’, ‘mobile application’, ‘E-health’ and ‘telemedicine’ (see Additional file 1 for full search strategy). Finally forward and backward citation tracking was conducted through Web of Science for all articles identified for full article review and one heavily cited review in this area [26]. One identified record was a ‘letter to the editor’ [36]. The corresponding author was contacted and confirmed that the data within the letter were also included in a further publication identified in the literature search [37]. In addition, the corresponding author of three articles written by the same author [38–40] was contacted who confirmed that all three publications included data collected from one single study. The data from only the largest study [40] were included in the final data synthesis to avoid data duplication. No supplementary data or further studies were identified by author contact.

### Study selection

Two researchers (EL, KH) screened articles for eligibility. Any discrepancies that could not be resolved were discussed with the other researchers (FD, RR). Studies were included if they a) administered an internet/web/online/computer intervention during the perinatal period, defined as the start of pregnancy to 1 year post-partum, b) had maternal mental health as an outcome measure and c) an assessment of mental health pre-and post-intervention. There were no exclusions related to study design or participants’ age, ethnicity, socioeconomic status, parity, depressive status or depression therapy.

### Data extraction

Relevant information on study characteristics and methodology (Table 1) and study results (Table 2) was extracted using pre-specified and standardised data extraction forms based on the template devised by the Cochrane Consumers and Communication Review Group [41]. One researcher (EL) completed the data extraction tables and a second researcher (KH) independently checked content. Any disagreements were discussed and resolved by consensus.

**Table 1:** Data extraction: Study design and details of intervention

First author	Year	Country	Study design	Participant number	Study population	Depressive status and treatment	Intervention	Comparator group	Intervention duration	Timing of intervention
Danaher [45]	2013	U.S. and Australia	FT with Quasi-experimental design	*n* = 53 (all received intervention)	Mean age of 31.9 years (SD 5.1), mean parity of 2 (SD 1.1), mean baby age of 5.5 months at pre-test (SD 2.9), 26 % graduate or higher level degrees.	All participants had EPDS of 12–20 or PHQ-9 of 10–19. 49 % (26/53) met DSM-IV criteria for MDD (SCID). No participants were undergoing current treatment for depression.	6 weekly online sessions, weekly phone calls from a personal coach plus automatic email reminders, private peer-based web forum, separate partner site	None	6–12 weeks	Post-natal
Kersting [40]	2013	Germany	RCT	*n* = 228 (TG 115, WLC 113)	228 participants, 92 % female, mean age of 34.18 years, mean of 9.93 months since losing a pregnancy at a mean gestation of 17.8 weeks.	Applicants with severely depressed mood/suicidal ideation (DSM-IV criteria) were excluded. No participants were currently receiving additional treatment.	10x 45-minute writing exercises assigned biweekly based on CBT. 3 treatment phases: self-confrontation, cognitive reappraisal, social sharing. Therapist contact with feedback and instruction twice per phase	WLC	5 weeks	Following loss of pregnancy
O’Mahen [43]	2013	UK	RCT	*n* = 910 (TG 462, TAU 448)	910 women, mean age of 32.3/32.2 (TG/TAU), with a child <12 months old. Varied socioeconomic status.	Inclusion criteria of EPDS >12. Participants were permitted to be currently receiving treatment (medical or psychological).	11x 40-minute online sessions completed weekly. Based on behavioural activation principles. Weekly email reminders with links to homework exercises. Optional weekly online ‘clinics’ with ‘real-time’ responses to questioning. Intervention-specific chat room.	TAU with access to Netmums general depression chat-room	15 weeks	Post-natal
O’Mahen [44]	2014	UK	RCT	*n* = 83 (TG 41, TAU 42)	83 women, > 18 years, vast majority Caucasian (Intervention = 92.6 % and TAU = 92.9 % Caucasian)	All women met DSM-IV criteria for MDD and had an EPDS of > 12.	12 online sessions with weekly telephone support sessions (20–30 mins) based on behavioural activation. The sessions involved interactive exercises and worked examples. Supplemented by other Netmums features; ‘meet a mum’ and moderated chat room.	TAU with access to Netmums general depression chat-room	12 weeks	Post-natal

*FT* Feasibility Trial, *DSM-IV* Diagnostic and Statistical Manual of Mental Disorders – 4^th^ edition, *MDD* Major Depressive Disorder, *SCID* Structured Clinical Interview for Disorders, *RCT* Randomized Controlled Trial, *IG* Intervention Group, *WLC* Waiting List Condition, *CBT* Cognitive Behavioural Therapy, *TAU* Treatment As Usual, *EPDS* Edinburgh Postnatal Depression Scale

**Table 2:** Data extraction: Outcomes

First author	Primary Outcome Measure	Other Outcome Measure(s)	Assessment measure(s) for depression/anxiety	Assessment time-points	Attrition and Adherence	Results	Limitations
Danaher [45]	Depressive symptoms, acceptability and feasibility	Automatic thoughts, dyadic adjustment, parenting sense of competence, self-efficacy	EPDS (only for pre-test screening) and HRSD, PHQ-9	Pre-test, Post-test (3 months) and follow-up (6 months), PHQ-9 during coach calls at 2 and 4 weeks additionally	All 6 sessions of the program were completed by 87 % (46/53) of participants. Posttest data were collected from 89 % of participants (47/53) with the exception of the HRSD (45/53, 85 %) and 6-month follow-up data were collected from 87 % of participants (46/53). Overall attrition was 13 % (7/53) from pretest to 6-month follow-up. Average of 5.6/6 sessions viewed.	PHQ-9 scores decreased from pretest (mean 12.6, SD 4.1) to posttest (mean 5.0, SD 4.4) and the 6-month follow-up (mean 4.2, SD 3.9) (*p* < 0.001) with large effects post-test (partial r = 0.77) and 6-month follow-up (partial r = 0.82). At pretest, 55 % (29/53) participants met PHQ-9 criteria for minor or major depression. At posttest, 90 % (26/29) no longer met these PHQ-9 criteria. HRSD scores also decreased from pretest (mean 16.9, SD 6.9) to posttest (mean 7.0, SD 5.6) and the 6-month follow-up (mean 6.6, SD 6.8). Changes from pretest were statistically significant (*P* < .001) with large effects at posttest (partial *r* = .75) and 6-month follow-up (partial *r* = .71).^a^	No comparator group, women were allowed to engage with other therapies (e.g., pharmacotherapy, counselling) during the trial and thus it is difficult to deduce individual effect of intervention, ‘coach’ reliant. Quasi-experimental design with small convenience sample Quality score: poor
Kersting [40]	Prolonged grief, PTSD	General psychopathology (including depression and anxiety)	ICG, BSI	Baseline, post-treatment and 3- month and 12-month follow up	86.1 % in the TG completed the intervention. WLC had a completion rate of 88.5 %. Dropouts were younger.	% of participants scoring > ICG-R cut-off for prolonged grief differed significantly at post-treatment (TG = 28.7 %, WLC = 47.8 %) Mean depression scores for TG were significantly decreased at post-treatment (1.19 → 0.61, t(114) = 7.98, *p* < 0.001) Same for anxiety (0.7 → 0.37). Depression scores continued to improve in follow-up measurements.	Heavily therapist reliant, well-educated sample, questionable relevance to perinatal depression, intensive – high level of participant engagement required. Male participants were included. Self-rating questionnaire to rate psychotherapy Quality score: intermediate
O’Mahen [43]	Feasibility, acceptability, depressive symptoms	None	EPDS	At sign-up to the trial and 15-weeks	18.9 % (172/910) completed the longer baseline questionnaires. The 15-week follow-up EPDS was completed by 39 % (181/462) in treatment group and 36 % in TAU (162/448). Fewer participants completed the acceptability questionnaires.	Improvement in depressive symptoms for 61.3 % (*n* = 111/181) of TG and 41.4 % (*n* = 67/162) for TAU group. When all non-respondents are counted as depressed the intervention is still favoured. 115/462 (intervention) vs 71/448 (TAU) were not depressed.	Extremely high attrition rates – follow-up EPDS was completed by less than 40 % in each group, only 1 measure of depressive symptoms, Imperfect intervention – women reported struggle ‘keeping up.’ Online Recruitment. Quality score: intermediate
O’Mahen [44]	Depression and anxiety, attrition and adherence	Work and social impairment, social support, postnatal bonding, health service utilization.	EPDS, GAD-7	Baseline, 17 weeks and 6 months post-treatment	86 % (71/83) completed EPDS at post-treatment and 71 % (59/83) at 3 month follow-up. Women completed an average of 8 (SD 4.5) telephone support sessions and 5.36 (SD = 4.62) online modules	Clinically significant improvement in depression scores in 62.2 % (*n* = 23/37) of TG compared to 29.4 % (*n* = 10/34) of TAU. Odds ratio = 0.26 (95 % CI 0.10–0.71) after adjustment for baseline EPDS scores. Large Cohen’s d^b^ effect sizes for EPDS (−0.87, 95 % CI −0.42 to −1.32) and GAD-7 (−0.59, 95 % CI −1.11 to −0.07).	Online sample recruitment might give a sample that is more accepting and responsive to internet-based therapy, unable to assess the impact of telephone support vs. web-modules, only 1 follow-up assessment point, not ethnically diverse sample. Quality score: good

*EPDS* Edinburgh Postnatal Depression Scale, *HRSD* Hamilton Rating Scale for Depression, *PHQ-9* Patient Health Questionnaire-9, *SD* Standard Deviation, *PTSD* Post-Traumatic Stress Disorder, *ICG* Inventory of Complicated Grief, *BSI* Brief Symptom Inventory (provides several indices including the global severity index of overall mental health and indices for the subscale of depression and anxiety), *TG* Treatment Group, *WLC* Waiting List Condition, *TAU* Treatment As Usual, *GAD-7* Generalised Anxiety Disorder Assessment – 7

^a^ The following is a discussion excerpt from this paper although no explicit data can be found within results. “Program use duration was not significantly associated with improvement in depression as measured by trajectories of the PHQ-9”

^b^Cohen’s *d* is defined as the difference between 2 means divided by a standard deviation for the data

### Quality assessment

Two researchers (EL, KH) independently assessed the risk of bias for each study as an assessment of study quality, using a modified tool [42] with objective criteria relating to sample population and recruitment, intervention, randomisation, blinding, comparator group, use of validated outcome measure, follow-up and data analysis. A paper could attain a maximum score of 8, a score of 1–3 indicating poor quality, 4–6 intermediate quality and 7–8 good quality.

### Data analysis and synthesis

Due to considerable study heterogeneity a descriptive synthesis of results was conducted. This included a description of the study design and sample studied, details of the intervention including mode and duration of delivery, and the outcomes including change in mood scores post intervention and at later follow-up as well as attrition rates.

## Results

### Study design and participants

**Fig. 1:**
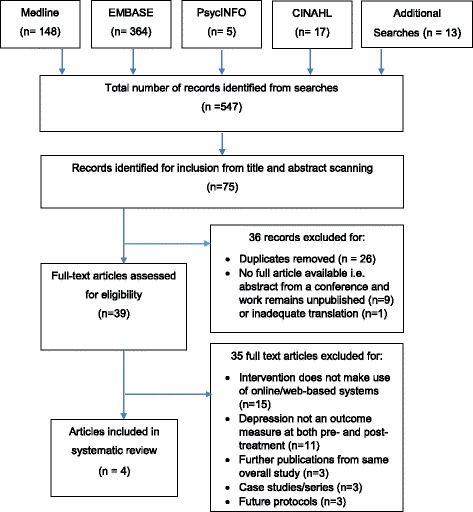
Flow (PRISMA) Diagram of Included Studies. Initial searches of bibliographic databases and reference tracking identified 547 records. Applying inclusion/exclusion criteria to the title and abstract identified 75 entries for full-article analysis. This was reduced to 39 following removal of duplicates and of records that did not have a corresponding full article. 4 studies were fully eligible for inclusion in the systematic review. Reasons for exclusion are outlined within the figure

From 547 abstracts, 39 full text articles were assessed (Fig. 1) and four studies were included in the final data synthesis. The four eligible studies included three randomised controlled trials (RCTs) [40, 43, 44] and one feasibility trial [45] (Tables 1 and 2) with data on 1274 participants from four countries. All studies included women ≥18 years of age with no history or current symptoms of severe or life-threatening mental illness, proficiency in the written language of the intervention and no participation in psychotherapy at recruitment. Those study participants who met criteria for depression ranged from ‘clinical impairment’ to ‘major depressive episode.’

### Intervention

The web-based intervention was administered postnatally in all four studies [40, 43–45], one of which recruited women with a recent loss of pregnancy [40]. None of the studies administered the intervention antenatally. Intervention duration ranged from 5 to 15 weeks with all four studies employing a modular structure. In addition to a web-based component, all studies included a therapist/external contact element to the intervention. In two studies [40, 43], contact with a therapist or other professional occurred through text-based contact i.e., email, instant messenger or chat-rooms whilst two studies included telephone sessions [44, 45]. Three studies provided their participants with access to a web-forum of the study cohort [43–45]. Studies differed in their reliance on an external support, with weekly feedback and monitoring proving a key element for some. In the three RCTs the comparator group were either assigned to treatment as usual or a waiting list condition.

### Outcomes

Depressive symptoms immediately post-intervention were a primary outcome for three of the studies [43–45] and a secondary outcome for the other [40]. Other primary outcomes included complicated grief and stress. All studies used well-recognised and validated scales for assessing depressive symptoms with two studies using the Edinburgh Postnatal Depression Scale (EPDS) [43, 44]. Three of the studies used a combination of outcome measures with one reporting findings from a single measure [43]. The EPDS score reductions in the two RCTs were (mean ± SEM) 8.52 ± 0.22 (Range 19.46 to 10.94) and 9.19 ± 0.63 (Range 20.24 to 11.05) for treatment groups and 5.16 ± 0.25 (Range 19.44 to 14.28) and 6.81 ± 0.71 (Range 21.07 to 14.26) for comparator groups. Three of the studies included further follow-up at a period ranging from 3 to 12 months post-completion of the intervention. The most common follow-up points were at 3 and 6 months post-treatment. Overall, all four studies described a benefit in maternal mood following intervention, either at post-treatment or follow-up.

Overall rates of attrition in the trials ranged from 13 to 61 % at the completion of the intervention assessment. However, the feasibility study [45] had extremely high attrition rates, with many women struggling to complete the intervention; excluding this study the early attrition ranged from 11 to 13 %. A common theme was the gradual increase in attrition as the study progressed, ranging at follow-up from 24 to 29 %. In one attrition was higher in younger women [40].

### Quality assessment

Quality assessment revealed one study to be of good quality, scoring 7 out of 8. Two studies were of intermediate quality (scoring 6 out of 8) where limitations included studying a selected sample (e.g., previous pregnancy loss [40], having child <12 months old [43] thus limiting the generalisability of findings to the general antenatal population. One study was categorised as poor. (Quality Assessment shown in Additional file 2).

## Discussion

Although CCBT has been endorsed by NICE for management of depressive symptoms in the general population, this systematic review demonstrates that there has been much less work in this field in relation to the perinatal period. We identified only four studies meeting the inclusion criteria for the review, three of which were RCTs. Importantly none reported an intervention delivered in the antenatal period, highlighting an important research gap. Though there were small numbers of studies with heterogeneity of study design, participant characteristics, duration, timing and nature of the intervention, and intermediate quality of studies, the overall findings suggest web-based interventions implemented in the post-partum period improve maternal mood outcomes.

In accord with other CCBT interventions, the majority of the interventions followed a modular structure. Most also included interaction with a therapist or other health professional as part of the intervention. This complicates the interpretation of the impact of the web-based component alone. Further, web-based interventions placing great importance on therapist input do not address the financial burden of treating perinatal depression nor the waiting list delay. In the case where web-based materials are used solely to supplement therapist mediated interventions, the many practical barriers to face-to-face contact may remain.

All studies utilised a well-validated outcome measure to assess depression symptoms. There was reduction in symptom scores in all studies, with the reported reduction in the two RCTs using the EPDS as an outcome measure, considered a clinically significant improvement [46, 47]. This review was not able to determine whether the severity of symptoms impacts on the effectiveness of CCBT. Three of the studies included longer-term follow-up assessments with the maximum follow-up at 12 months. Due to the relapsing and remitting course of depression, longer-term outcomes are important to evaluate in future studies.

Attrition rates are often high and problematic in other studies of online therapies [48, 49]. Of note the one feasibility study also had high attrition rates (less than 40 % of women completing the follow-up EPDS questionnaire). However, early attrition rates in the RCTs was much lower, and comparable between studies, though notably did increase with longer-term follow-up. There was insufficient data to determine whether attrition rates were related to length or mode of intervention, or whether this differed according to the level of support given during the intervention. Further work is needed to understand the reason for this in order to gain a comprehensive understanding of the true acceptability of CCBT [50]. It is also unknown whether use of a general intervention, rather than an intervention tailored to specific concerns associated with the post-partum period or event triggering the onset of depressive symptoms impacted attrition rates. Future studies should consider use of tailored interventions specific to pregnancy, the post-partum period and pregnancy specific triggering events (e.g., antenatal stillbirth). To understand and limit attrition, future studies should also consider detailed qualitative assessment of reasons for completing the intervention or not, as well as acceptability of the intervention [51], and consideration of co-morbid anxiety as well as depression symptoms.

The strengths of this review include the systematic and comprehensive review process which was followed in line with PRISMA guidelines. Two researchers independently assessed eligibility of the titles, abstracts and full-text studies, extracted the data and assessed the articles for bias. There are also a number of potential limitations. The overall number of studies identified was small and none were in the antenatal period. Whilst this highlights the need for more research in this field, it does limit the interpretation of the findings. Indeed the heterogeneity of study design and lack of consistency in outcome measures meant we were unable to quantitate outcomes. Two of the studies included selected women, and one study recruited both women and their partners, limiting generalisability of the findings. Some of the studies included women with pre-existing depression or taking antidepressant therapy which may have confounded the results with treatment effects. Finally lack of information about acceptability of the interventions was a limitation of these studies.

This review highlights a number of directions for future research. More work is both warranted and necessary within the neglected area of E-health for antenatal depression. Antenatal depression has received less attention than postpartum depression, despite a similar prevalence. This is important, as antenatal depression is a major risk factor for depression in the postpartum period. Thus an intervention administered during pregnancy may also prevent the development and/or progression of antenatal depression to postpartum depression following childbirth. Whilst there is preliminary evidence that CCBT interventions are acceptable during pregnancy [51], further studies are needed to investigate the impact of such an intervention during pregnancy [33, 52, 53]. Our review would also suggest there is a need for more rigor in this field, particularly harmonisation of symptom scores used to allow future meta-analyses of outcomes. Whilst our review focused on a maternal intervention, consideration of an intervention including the partner and/or family may be an important approach for future work, particularly when the triggering event (e.g., fetal death, significant life event) is likely to affect both the woman and her partner.

## Conclusions

In summary, this systematic review suggests web-based interventions may be effective for the management of maternal health in the post-partum period and highlights the need for robust, high quality studies conducted during the antenatal period.

## Additional files

Additional file 1: Systematic Search Strategies. Full search strategies of all databases used within the systematic review. (PDF 107 kb) Additional file 2: Quality Assessment. Quality assessment by each researcher (EL/KH) of non-case-based studies. (PDF 52 kb)
