# Supplementary material for: Web-based interventions for prevention and treatment of perinatal mood disorders: a systematic review
Source: BMC Pregnancy Childbirth. 2016 Feb 29;16:38. doi: 10.1186/s12884-016-0831-1 (PMC4770541; doi:10.1186/s12884-016-0831-1)
Supplement: Additional file 1: — Systematic Search Strategies. Full search strategies of all databases used within the systematic review. (PDF 107 kb) [file 12884_2016_831_MOESM1_ESM.pdf]

Additional file 1: Database search strategies

**Ovid MEDLINE(R)** 1946 to March Week 2 2015			
**#**	**Searches**	**Results**	**Search Type**
1	Depression/	79319	Advanced
2	(depressive or depressed).mp. or depression.ti,ab. [mp=title, abstract, original title, name of substance word, subject heading word, keyword heading word, protocol supplementary concept word, rare disease supplementary concept word, unique identifier]	299894	Advanced
3	Depression, Postpartum/	3533	Advanced
4	1 or 2 or 3	321197	Advanced
5	Pregnancy/	704364	Advanced
6	Perinatal Care/	2939	Advanced
7	Prenatal Care/	20722	Advanced
8	Postnatal Care/	4008	Advanced
9	Pregnancy Complications/	74677	Advanced
10	(ante?natal or peri?natal or pre?partum or post?natal).mp. or post?partum.ti,ab. [mp=title, abstract, original title, name of substance word, subject heading word, keyword heading word, protocol supplementary concept word, rare disease supplementary concept word, unique identifier]	170329	Advanced
11	5 or 6 or 7 or 8 or 9 or 10	798167	Advanced
12	4 and 11	13736	Advanced
13	Self Care/	24322	Advanced
14	Self-Help Groups/	7907	Advanced
15	Behaviour Therapy/ or Cognitive Therapy/	39427	Advanced
16	Internet/	51499	Advanced
17	Psychotherapy/ or Therapy, Computer-Assisted/ or Software/ or Online Systems/	133824	Advanced
18	(mobile applications or software design or user-computer interface or video games or web browser).mp. [mp=title, abstract, original title, name of substance word, subject heading word, keyword heading word, protocol supplementary concept word, rare disease supplementary concept word, unique identifier]	36037	Advanced
19	(self?care or self?help or self?management or intervention* or web*).mp. or E?health.ti,ab.	619523	Advanced

	[mp=title, abstract, original title, name of substance word, subject heading word, keyword heading word, protocol supplementary concept word, rare disease supplementary concept word, unique identifier]		
20	Telemedicine/	11775	Advanced
21	Humans/	13760465	Advanced
22	16 or 17 or 18 or 19 or 20	790204	Advanced
23	13 or 14 or 15	69969	Advanced
24	4 and 11 and 21 and 22 and 23	148	Advanced

**EMBASE** 1980 to 2015 Week 11			
**#**	**Searches**	**Results**	**Search Type**
1	depression/	252808	Advanced
2	depressive.mp. or depressed.ti,ab. [mp=title, abstract, subject headings, heading word, drug trade name, original title, device manufacturer, drug manufacturer, device trade name, keyword]	177234	Advanced
3	mental health/	78875	Advanced
4	wellbeing/	36190	Advanced
5	puerperal depression/	6400	Advanced
6	1 or 2 or 3 or 4 or 5	445846	Advanced
7	pregnancy/	526890	Advanced
8	perinatal care/	10491	Advanced
9	prenatal care/	27485	Advanced
10	postnatal care/	5021	Advanced
11	puerperium/	27267	Advanced
12	pregnancy complication/	69165	Advanced
13	(ante?natal or peri?natal or pre?partum or post?natal).mp. or post?partum.ti,ab. [mp=title, abstract, subject headings, heading word, drug trade name, original title, device manufacturer, drug manufacturer, device trade name, keyword]	231155	Advanced
14	7 or 8 or 9 or 10 or 11 or 12 or 13	716340	Advanced
15	self care/	33579	Advanced
16	self help/	11563	Advanced
17	behaviour therapy/	37114	Advanced
18	cognitive therapy/	35758	Advanced

19	psychotherapy/ or psychodynamic psychotherapy/	76653	Advanced
20	cognitive behavioural therapy.mp. or cognitive therapy/	36173	Advanced
21	Internet/	77807	Advanced
22	online system/ or online therapy.mp.	18204	Advanced
23	computer aided design/ or computer/ or computer assisted diagnosis/ or computer program/	257326	Advanced
24	(mobile applications or software design or user-computer interface or video games or web browser).mp. [mp=title, abstract, subject headings, heading word, drug trade name, original title, device manufacturer, drug manufacturer, device trade name, keyword]	6217	Advanced
25	telemedicine/	12141	Advanced
26	(intervention* or web*).mp. or E?health.ti,ab. [mp=title, abstract, subject headings, heading word, drug trade name, original title, device manufacturer, drug manufacturer, device trade name, keyword]	906209	Advanced
27	15 or 16 or 17 or 18 or 19 or 20	170103	Advanced
28	21 or 22 or 23 or 24 or 25 or 26	1203502	Advanced
29	6 and 14 and 27 and 28	367	Advanced
30	limit 29 to human	363	Advanced

**CINAHL SEARCH – 20/3/15**	
1	(MH "Anxiety") OR (MH "Depression")
2	depressed or depressive. TI, AB
3	(MH "Mental Health")
4	(MH "Psychological Well-Being")
5	(MH "Mindfulness")
6	(MH "Depression, Postpartum")
7	1 OR 2 OR 3 OR 4 OR 5 OR 6
8	(MH "Pregnancy")
9	(MH "Prenatal Care") OR (MH "Prenatal Care (Iowa NIC)")
10	(MH "Perinatal Care")
11	(MH "Postnatal Care") OR (MH "Postpartum Care (Saba CCC)")
12	(MH "Pregnancy Complications")

13	(MH "Pregnancy Complications, Psychiatric")
14	8 OR 9 OR 10 OR 11 OR 12 OR 13
15	(MH "Self Care")
16	(MH "Cognitive Therapy (Iowa NIC) (Non-Cinahl)") OR (MH "Cognitive Therapy")
17	(MH "Behaviour Therapy") OR (MH "Behaviour Therapy (Iowa NIC) (Non-Cinahl)") OR (MH "Behaviour Modification")
18	(MH "Psychotherapy") OR (MH "Psychotherapy, Brief")
19	15 OR 16 OR 17 OR 18
20	(MH "Therapy, Computer Assisted")
21	(MH "Internet") OR (MH "Telepsychiatry")
22	(MH "Telemedicine") OR (MH "Telehealth")
23	(MH "World Wide Web") OR (MH "Website Development") OR (MH "World Wide Web Applications")
24	(MH "Online Services") OR (MH "Online Systems")
25	(MH "User-Computer Interface")
26	"e-health"
27	"Web*"
28	20 OR 21 OR 22 OR 23 OR 24 OR 25 OR 26 OR 27
29	7 AND 14 AND 19 AND 28

**PsycINFO** 1987 to March Week 3 2015			
**#**	**Searches**	**Results**	**Search Type**
1	"depression (emotion)"/	10740	Advanced
2	depressive.mp. or depressed.ti,ab. [mp=title, abstract, heading word, table of contents, key concepts, original title, tests & measures]	89863	Advanced
3	mental health/	38055	Advanced
4	postpartum depression/	3110	Advanced
5	1 or 2 or 3 or 4	132579	Advanced
6	pregnancy/	14122	Advanced
7	prenatal care/	1363	Advanced
8	perinatal period/	1813	Advanced
9	postnatal period/	3254	Advanced

10	(ante?natal or peri?natal or pre?partum or post?natal).mp. or post?partum.ti,ab. [mp=title, abstract, heading word, table of contents, key concepts, original title, tests & measures]	26226	Advanced
11	7 or 8 or 9 or 10	27111	Advanced
12	self care skills/	2952	Advanced
13	self management/	4437	Advanced
14	self help techniques/	3049	Advanced
15	cognitive behaviour therapy/	12498	Advanced
16	cognitive therapy/	10805	Advanced
17	behaviour therapy/	7413	Advanced
18	psychotherapy/	34649	Advanced
19	relaxation therapy/	1466	Advanced
20	12 or 13 or 14 or 15 or 16 or 17 or 18 or 19	72618	Advanced
21	internet/	22999	Advanced
22	websites/	3378	Advanced
23	computer assisted therapy/	573	Advanced
24	online therapy/	1626	Advanced
25	(software or online systems or mobile applications or software design or user-computer interface or video games or web browser).mp. [mp=title, abstract, heading word, table of contents, key concepts, original title, tests & measures]	21464	Advanced
26	telemedicine/	2764	Advanced
27	web*.mp.	33157	Advanced
28	21 or 22 or 23 or 24 or 25 or 26 or 27	69690	Advanced
29	5 and 11 and 20 and 28	5	Advanced
30	limit 29 to human	5	Advanced

Additional file 1: Clinicaltrials.gov was searched on the 26th of March 2015 by combination of a number of key terms. All searches were conducted using the advanced searcher.

Search 1: “online” AND “therapy” AND “depression” AND “pregnancy
(3 results)

Search 2: “web-based” AND “intervention” AND “depression” AND “pregnancy”
(4 results)

Search 3: “web-based” AND “therapy” AND “depression” AND “pregnancy”
(4 results)

Search 4: “online” AND “therapy” AND “mental-health” AND “perinatal”
(1 result)

Search 5: “online” AND “therapy” AND “depression” AND “post-partum”
(3 results)

Search 6: “online” AND “CBT” AND “depression” AND “pregnancy”
(2 results)

Search 7: “computerized cognitive behavioural therapy” AND “pregnancy”
(0 results)

Search 8: “online” AND “cognitive therapy” AND “depression” AND “pregnancy”
(2 results)

Search 9: “internet” AND “therapy” AND “depression” AND “pregnancy”
(9 results)

Search 10: “Internet: AND “intervention” AND “depression” AND “pregnancy”
(13 results)

Search 11: “Internet: AND “intervention” AND “depression” AND “post-partum”
(11 results)

Search 12: “Internet: AND “intervention” AND “mental-health” AND “post-partum”
(6 results)

Search 13: “Computer-assisted” AND “depression” AND “pregnancy”
(1 result)

Search 14: “Computer-assisted” AND “depression”
(16 results)
