# Supplementary material for: Web-based interventions for prevention and treatment of perinatal mood disorders: a systematic review
Source: BMC Pregnancy Childbirth. 2016 Feb 29;16:38. doi: 10.1186/s12884-016-0831-1 (PMC4770541; doi:10.1186/s12884-016-0831-1)
Supplement: Additional file 2: — Quality Assessment. Quality assessment by each researcher (EL/KH) of non-case-based studies. (PDF 52 kb) [file 12884_2016_831_MOESM2_ESM.pdf]

Additional File 2: Completed quality-scoring table (EL)

**Study**	Study population characteristics/size /recruitment (appropriate?)	Specifically adapted interven-tion?	Random-isation?	Blinding?	Comparator group?	Validated outcome measure?	Follow-up?	Clear findings with appropriate analysis?	**Total Score**
**Danaher 2013**	0	1	0	0	0	1	1	0	**3**
**Kersting 2013**	0	1	1	0	1	1	1	1	**6**
**O`Mahen 2013**	1	1	1	0	1	1	0	1	**6**
**O`Mahen 2014**	1	1	1	0	1	1	1	1	**7**

Each paper was given a score out of 8 as evaluated by 2 independent researchers. ‘1’ means ‘yes’ and that the paper satisfies that criterion and ‘0’ means ‘no’, that the paper has not. The total score is the total number of criteria satisfied. A score of 1-3 means the paper is of poor quality, 4-6 is of intermediate quality and a score of 7-8 denotes a paper of good quality

Additional file 2: Completed quality-scoring table (KH)

**Study**	Study population characteristics/size /recruitment (appropriate?)	Specifically adapted interven-tion?	Random-isation?	Blinding?	Comparator group?	Validated outcome measure?	Follow-up?	Clear findings with appropriate analysis?	**Total Score**
**Danaher 2013**	1	0	0	0	0	1	0	1	**3**
**Kersting 2013**	0	1	1	0	1	1	1	1	**6**
**O`Mahen 2013**	0	1	1	0	1	1	1	1	**6**
**O`Mahen 2014**	1	1	1	0	1	1	1	1	**7**

Each paper was given a score out of 8 as evaluated by 2 independent researchers. ‘1’ means ‘yes’ and that the paper satisfies that criterion and ‘0’ means ‘no’, that the paper has not. The total score is the total number of criteria satisfied. A score of 1-3 means the paper is of poor quality, 4-6 is of intermediate quality and a score of 7-8 denotes a paper of good quality
